# Benchmarking the topological accuracy of bacterial phylogenomic workflows using *in silico* evolution

**DOI:** 10.1099/mgen.0.000799

**Published:** 2022-03-15

**Authors:** Boas C. L. van der Putten, Niek A. H. Huijsmans, Daniel R. Mende, Constance Schultsz

**Affiliations:** ^1^​ Department of Medical Microbiology, Amsterdam UMC, University of Amsterdam, Amsterdam, The Netherlands; ^2^​ Department of Global Health, Amsterdam Institute for Global Health and Development, Amsterdam UMC, University of Amsterdam, Amsterdam, The Netherlands

**Keywords:** benchmarking study, *in silico *evolution, phylogenetics, simulation

## Abstract

Phylogenetic analyses are widely used in microbiological research, for example to trace the progression of bacterial outbreaks based on whole-genome sequencing data. In practice, multiple analysis steps such as *de novo* assembly, alignment and phylogenetic inference are combined to form phylogenetic workflows. Comprehensive benchmarking of the accuracy of complete phylogenetic workflows is lacking. To benchmark different phylogenetic workflows, we simulated bacterial evolution under a wide range of evolutionary models, varying the relative rates of substitution, insertion, deletion, gene duplication, gene loss and lateral gene transfer events. The generated datasets corresponded to a genetic diversity usually observed within bacterial species (≥95 % average nucleotide identity). We replicated each simulation three times to assess replicability. In total, we benchmarked 19 distinct phylogenetic workflows using 8 different simulated datasets. We found that recently developed *k*-mer alignment methods such as kSNP and ska achieve similar accuracy as reference mapping. The high accuracy of *k*-mer alignment methods can be explained by the large fractions of genomes these methods can align, relative to other approaches. We also found that the choice of *de novo* assembly algorithm influences the accuracy of phylogenetic reconstruction, with workflows employing SPAdes or skesa outperforming those employing Velvet. Finally, we found that the results of phylogenetic benchmarking are highly variable between replicates. We conclude that for phylogenomic reconstruction, *k*-mer alignment methods are relevant alternatives to reference mapping at the species level, especially in the absence of suitable reference genomes. We show *de novo* genome assembly accuracy to be an underappreciated parameter required for accurate phylogenomic reconstruction.

## Impact Statement

Phylogenetic analyses are crucial to understand the evolution and spread of microbes. Among their many applications is the reconstruction of transmission events, which can provide information on the progression of pathogen outbreaks; for example, to investigate foodborne outbreaks such as the 2011 outbreak of *Escherichia coli* O104:H4 across Europe. As different microbes evolve differently, it is important to know which phylogenetic workflows are most accurate when working with diverse bacterial data. However, benchmarks usually consider only a limited dataset. We therefore employed a range of simulated evolutionary scenarios and benchmarked 19 phylogenetic workflows on these simulated datasets. An advantage of our simulation approach is that we know *a priori* what the outcome of the analyses should be, allowing us to benchmark accuracy. We found significant differences between phylogenetic workflows and were able to dissect which factors contribute to phylogenetic analysis accuracy. Taken together, this new information will hopefully enable more accurate phylogenetic analysis of bacterial outbreaks.

## Data Summary

A Zenodo repository is available at https://doi.org/10.5281/zenodo.5036179 containing all the simulated genomes, all alignments produced by phylogenetic workflows and .csv files summarizing the topological accuracies of phylogenies produced based on these alignments. Code is available at https://github.com/niekh-13/phylogenetic_workflows.

## Introduction

Phylogenetic analyses are crucial to assess the relatedness within a population of micro-organisms. These analyses provide information on the speciation, evolution and spread of microbes. Within clinical settings, they can be used to identify microbial outbreaks and transmission events [1]. With the introduction of cost-efficient whole-genome sequencing, bacterial outbreak tracing is increasingly based on whole-genome data, instead of on a small section of the genome such as 16S rRNA genes or a set of universal genes [2]. Whole-genome phylogenetic analysis can be applied by various pipelines or workflows, often composed of multiple separate tools. Common differences between workflows are which genomic loci are considered in the analysis (only protein-encoding genes or also intergenic regions), how genetic features are defined (genes, *k*-mers, single nucleotide variants, etc.), but also how genomes are assembled. Benchmarking is necessary to make sense out of the plethora of bioinformatic methodologies available. Although previous benchmarks of bacterial phylogenetic reconstruction have generated important insights [3–5], some gaps remain. For example, the usefulness of recently developed *k*-mer alignment methods has not been fully explored in previous benchmark exercises. Additionally, the role of using different *de novo* assembly methods prior to comparative analysis has received little attention (especially in combination with the aforementioned *k*-mer alignment methods). Other methodological choices (e.g. choice of phylogenetic tree inference) have been amply studied before [3].

Benchmarking phylogenetic workflows requires knowledge of the true phylogenetic tree, as benchmarking results need to be compared to this reference. The true phylogenetic tree is typically not known in real-world settings. As such, various approaches have been proposed to determine or estimate the true phylogenetic tree of a set of strains. Some previous studies have assumed that the consensus of all phylogenies produced by the studied methods is close to the true phylogeny. Alternatively, studies have collated benchmark data sets where the epidemiological data was concordant with the phylogenomic analyses [4]. Because this approach uses real-life data, little is known about the underlying genetic events, and it does not allow one to experimentally vary evolutionary parameters. Another approach is to have a mutant strain with an increased mutation rate evolve *in vitro*, and determine the structure of the true phylogeny from the experimental evolution controlled in the lab [5]. This approach provides a good grasp of the true phylogeny and allows the sampling of ancestral strains, but the method is costly and time-consuming, and evolutionary parameters cannot be easily controlled. Finally, some studies have used *in silico* evolution to produce realistic sequencing data together with an *a priori* defined true phylogeny [3, 4, 6–9]. This approach offers the possibility to increase or decrease the rate of a range of evolutionary events, such as point mutations, indels, gene duplication, gene loss, gene translocation and lateral gene transfer. Additionally, genomic regions can be evolved under different evolutionary models, as is typical in real-life scenarios (e.g. protein-encoding genes vs intergenic regions). Finally, this approach allows a comparison to the true phylogeny, which is not possible with other methods.

Several *in silico* evolution frameworks have been developed, with differing goals and strengths [3, 7–13]. In the current study, we aimed to select a simulation strategy producing complete, haploid bacterial genomes. As lateral gene transfer is a common phenomenon in bacteria, simulation of lateral gene transfer should be included during *in silico* evolution. As we aimed to compare against a true tree, the *in silico* evolution was guided by a user-provided phylogenetic tree. We surveyed the Genetic Data Simulator database (https://surveillance.cancer.gov/genetic-simulation-resources/) and previously published manuscripts [3, 4, 7–11, 13]. The workflow used by Lees *et al*. (2018) [3] was used as it satisfied all our criteria. The workflow combines alf and dawg software, and enables easy tuning of evolutionary parameters and setting simulation seeds for reproducible analysis.

In this study, our aim was to assess which bioinformatic workflows are able to reconstruct the true phylogeny accurately under diverse evolutionary scenarios. We consider simulating evolution *in silico* to be the optimal approach to achieve this. We simulated the evolution of *Escherichia coli* genomes *in silico* under eight different scenarios, varying the rates of indels, gene duplication, gene loss and lateral gene transfer. We used these simulated datasets to assess the topological accuracy of 19 phylogenetic reconstruction workflows, including *de novo* genome assembly, alignment or mapping, and finally phylogenetic tree inference. We included six alignment or mapping methods to identify SNPs between samples, which can be subdivided into *k*-mer alignment, reference mapping and gene-by-gene alignment methods. We also included three different *de novo* assembly approaches, as the impact of this pre-processing step on phylogenomic accuracy is understudied.

## Methods

### Study design

**Fig. 1.**
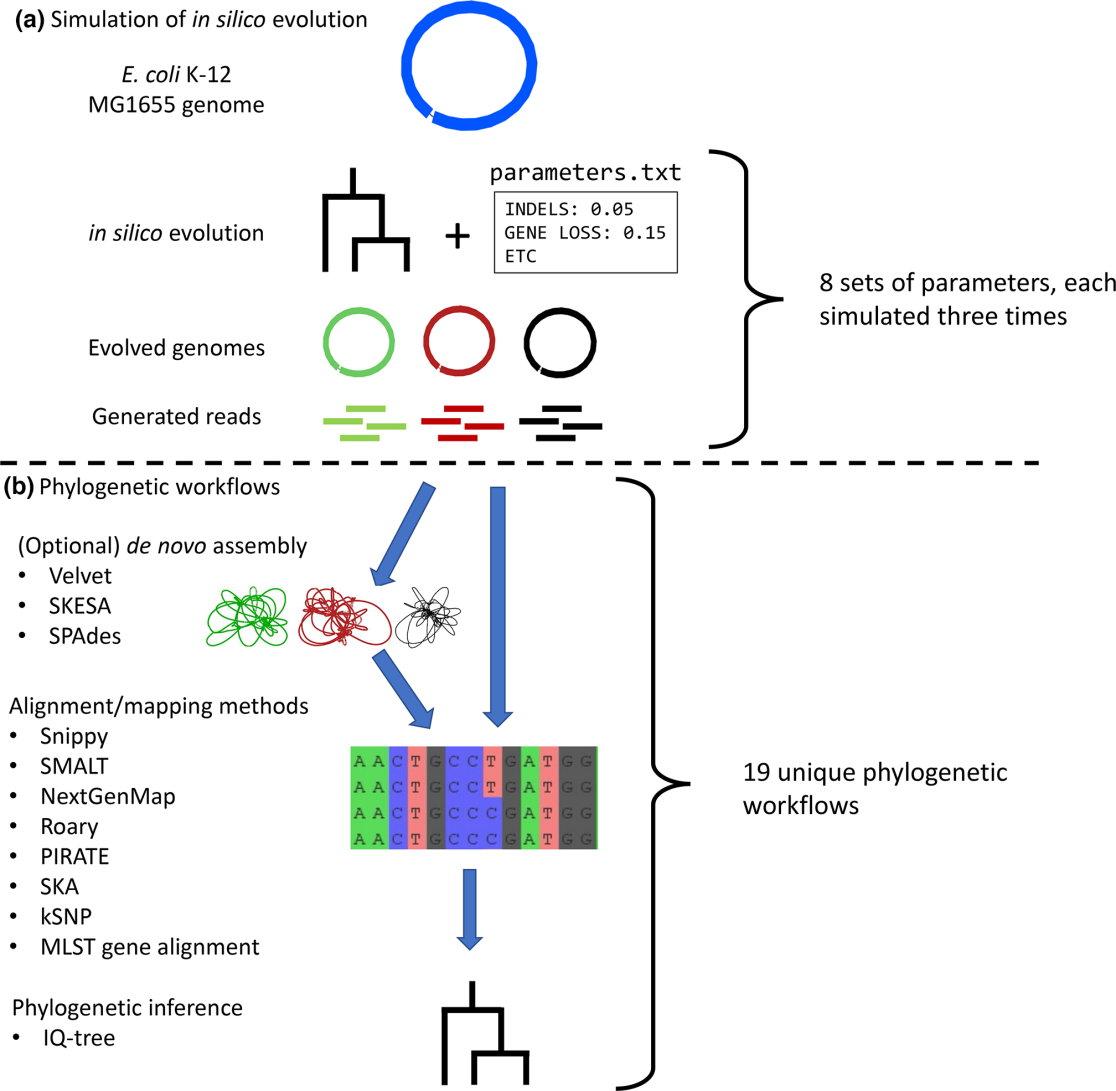
Overview of this study. (**a**) Simulation of the *in silico* evolution. The *E. coli* K-12 MG1655 genome is evolved *in silico* according to a phylogeny (providing genetic distances) and a set of parameters controlling the rates of genetic events (providing which genetic events result in the genetic distance provided by the phylogeny). The resulting genomes are depicted by coloured complete genome graphs visualized in Bandage [47]. The complete genomes are subsequently shredded into sequencing reads. (**b**) Phylogenetic workflows. Generated sequencing reads are assembled into draft genomes (coloured draft genome graphs) or directly mapped onto the ancestral genome. From alignments, phylogenetic trees are inferred using iq-tree.

This study consists of two main parts: simulation of *in silico* genome evolution (Fig. 1a) and application of phylogenetic workflows on the simulated data sets (Fig. 1b). A total of eight sets of parameters were used to simulate a variety of evolutionary processes on genic and intergenic regions separately, using the same phylogeny every time (Table S1, available with the online version of this article). Each simulation was repeated three times with different random seeds to obtain technical replicates. From the *in silico* evolved genomes, short sequencing reads were generated. These sequencing reads were then used as input for the 17 phylogenetic workflows. We tested three *de novo* assembly algorithms in the workflows (Velvet, skesa, SPAdes), alongside two methods for core-gene alignment (Roary, pirate), one method for multilocus sequence typing (MLST) gene alignment (mlst-check), two methods for *k*-mer alignment (ska, kSNP) and three methods for reference-based read mapping that performed well in a recent benchmark [14] (Snippy, NextGenMap, smalt). A total of 19 phylogenetic workflows were tested (Table S2). All phylogenies have been inferred from alignments using iq-tree and ModelFinder. As the same phylogeny was used for each simulation, but the parameters for genetic events changed between simulations, each simulated dataset is expected to yield the same genetic distance between isolates (governed by the phylogeny), although the genetic events that have led to this identical genetic distance could be different (governed by the parameters).

### *In silico* evolution

All code is available as a Snakemake v5.8.1 [15] pipeline at https://github.com/niekh-13/phylogenetic_workflows. All tools were run using default parameters, unless otherwise noted. The complete chromosome of *E. coli* K-12 MG1655 (RefSeq assembly GCF_000005845.2) was used as the ancestral genome in all simulations. Evolution was simulated according to the phylogeny described by Kremer *et al*. [16]. The general approach used in this study was based on the approach described by Lees *et al*. [3]. The ancestral genome was annotated using Prokka v1.14.6 [17] and subsequently divided into protein-encoding genes and intergenic regions (all sequences not annotated as protein-encoding gene). Protein-encoding regions were *in silico* evolved using Artificial Life Framework v1.0 (alf) [12], while intergenic regions were *in silico* evolved using dawg v2.0.beta1 [13].

alf simulations were run using an empirical codon model, using a standard indel rate of 0.0252, a gene duplication and gene loss rate of 0.05, lateral gene transfer rates of 0.04 for single genes and 0.16 for groups of genes, and no spontaneous gene inversion or gene translocation, based on previous bacterial simulations [3]. Complete specifications for the default run are available from https://github.com/niekh-13/phylogenetic_workflows/blob/master/input/alf_protein_sim.drw. Seven additional simulations were performed (Table S1): ‘Indel × 0.5’ (halved indel rate), ‘Indel × 2’ (doubled indel rate), ‘gene duplication × 2’ (doubled gene duplication rate), ‘gene loss × 2’ (doubled gene loss rate), ‘gene duplication × 2 and gene loss × 2’ (doubled gene duplication and gene loss rates), ‘lateral gene transfer × 0.5’ (halved lateral gene transfer rate for single genes and groups of genes), ‘lateral gene transfer × 2’ (doubled lateral gene transfer rate for single genes and groups of genes).

dawg simulations were run using a default indel rate of 0.00175 and evolved under a general time-reversible (GTR) model with rates A↔C, 0.91770; A↔G, 4.47316; A↔T, 1.10375; C↔G, 0.56499; C↔T, 6.01846; G↔T, 1.00000; based on the GTR matrix inferred from a dataset of nearly 1200 *E. coli* strains isolated from various host species (HECTOR study, unpublished results). For simulations indel × 0.5 and indel × 2, the indel rate was changed appropriately (Table S1).

Per simulation, alf and dawg *in silico* evolution yielded protein-encoding genes and intergenic regions for 96 *in silico* evolved genomes. These were assembled into 96 complete genomes. As stop codons are removed during alf simulation, stop codons were inserted at the ends of genes. Paired-end sequencing reads in fastq format were simulated using art v2016.06.05 [18], based on an Illumina HiSeq 2500 profile with 30× depth, read length of 150 bp and a mean DNA fragment size of 600 bp with a standard deviation of 10 bp, using seed 21 (flags ‘-ss HS25 -na -rs 21 p -l 150 f 30 m 600 s 10’).

For the generation of clonal datasets, we divided branch lengths of the true tree by factor 3, 30 and 100 corresponding to a median average nucleotide identity (ANI) of 99.0, 99.5 and 99.9 % between genomes, respectively. Clonal datasets were generated using the standard rates for indel, gene duplication, gene deletion and lateral gene transfer events.

### Comparing pipelines

From the simulated Illumina sequencing reads, phylogenies were reconstructed through 19 workflows (Table S2). Assemblies were created using the Shovill v1.1.0 (https://github.com/tseemann/shovill) wrapper for Velvet v1.2.10 [19], SPAdes v3.14.0 using ‘--isolate’ mode [20] and skesa v2.3.0 [21]. Contigs were retained if they were 500 bp or larger for all *de novo* assembly algorithms. Assembly quality metrics were assessed using Quast v5.0.2 [22] and all-versus-all ANI comparisons were made using fastANI v1.2 [23]. *k*-mer alignment methods kSNP v3.1 [24] and ska v1.0 [25] were used on all assemblies, and ska was additionally run on sequencing reads. In our study, both tools were used to extract *k*-mers of 31 bp from assemblies or sequencing reads. Subsequently, these tools aligned *k*-mers of which the first and last 15 bp were identical; thus, allowing only the middle base to vary between aligned *k*-mers. This *k*-mer alignment produced SNP alignments, which can be used for phylogenetic inference. Important to note is that although ska and kSNP also employ *k*-mer-based methods, these methods are conceptually distinct from other *k*-mer-based tools such as Mash (https://github.com/marbl/Mash). The mapping pipelines Snippy v4.6.0 (https://github.com/tseemann/snippy), NextGenMap v0.5.5 [26] and smalt v0.7.6 [27] were used on sequencing reads alone, using the *E. coli* K-12 MG1655 chromosome as a reference (RefSeq assembly GCF_000005845.2). As all genomes in the current study are simulated from this chromosome, this represents the most suitable reference. Gene-by-gene methods Roary v3.13.0 [28] and pirate v1.0.3 [29] were used on annotations produced by Prokka v1.14.6. Finally, alignments were constructed from MLST genes using mlst-check v2.1.1706216 [30] and realigned using ClustalO v1.2.4 [31]. All methods, including *k*-mer alignment methods, produce nucleotide alignments, which were subsequently used to infer phylogenies using iq-tree v2.0.3 [32] and ModelFinder [33] packaged with iq-tree. Differences between the ground truth phylogeny and produced phylogenies were assessed using the Robinson–Foulds distance calculation implemented in ape v5.4 [34] and the Kendall–Colijn distance calculation implemented in treespace v1.1.3.2 [35]. All simulations and pipelines were run three times, with seeds 1, 42 and 1704 in the alf simulation. Alignment lengths were extracted using snp-sites v2.5.1 [36].

### Visual and statistical analysis

Parsing of results was performed using the pandas library v0.25.3 [37] in Python v3.8.3, and using the tidyverse v1.3.0 [38] and rstatix v0.6.0 (https://cran.r-project.org/package=rstatix) libraries in R v4.0.1. Results were plotted using ggplot2 v3.3.1 [39], ggpubr v0.4.0 (https://cran.r-project.org/package=ggpubr), ggthemes v4.2.0 [40], patchwork v1.0.1 [41] and using SuperPlotsOfData [42]. Tests for statistical significance were carried out using the scipy library [43] using paired Wilcoxon ranked sum tests where indicated. Bonferroni correction for multiple testing was applied where applicable.

## Results

### Reference-based mapping and *k*-mer alignment methods yield phylogenetic trees most similar to ground truth

The *in silico* evolution yielded isolate sets with a genetic diversity comparable to a single bacterial species (≥95 % ANI [44]; Figs 1 and S1). The same level of genetic diversity was attained between simulations, although these simulations included different rates of simulated genetic events (substitutions, indels, lateral gene transfer, etc.; Table S3).

The optimal phylogenetic workflow should produce a phylogeny identical to the one that was used in the simulation process (the ground truth phylogeny). Per workflow, we calculated tree distance between the phylogeny produced by the workflow and the ground truth phylogeny. Tree distances were expressed in the Robinson–Foulds distance and the Kendall–Colijn metric.

**Fig. 2.**
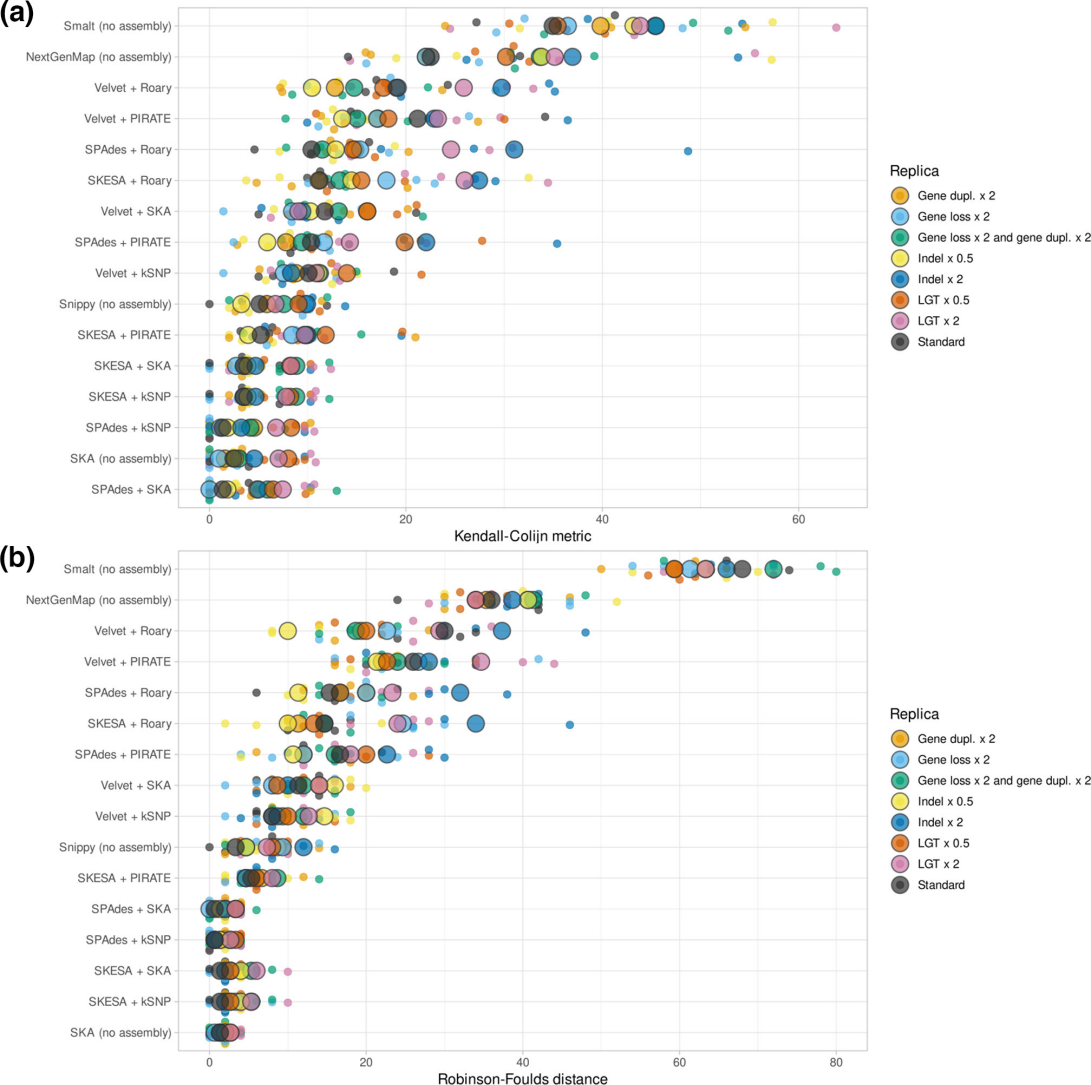
Kendall–Colijn metrics and Robinson–Foulds distances per phylogenetic workflow across eight simulations. Displayed distances are calculated between the ground truth phylogeny and the phylogeny produced by the relevant workflow. Generated using SuperPlotsOfData, and ordered by median. Large circles indicate the median of replicates. Small circles indicate separate measurements for a replica.

The workflow showing the lowest tree distances across simulations employed SPAdes *de novo* assembly and subsequently ska for *k*-mer alignment. After Bonferroni correction for multiple testing, the Kendall–Colijn metric of this workflow was significantly lower than all other workflows except Snippy, SPAdes+kSNP, skesa+kSNP, and skesa+ska (Fig. 2, Table S4). Notably, core-gene-alignment methods and methods employing Velvet for *de novo* assembly performed worse in our study. MLST gene alignment methods showed the highest deviation from the ground truth phylogeny as measured by Kendall–Colijn metric and Robinson–Foulds distance (Fig. S2).

We also simulated more clonal datasets with a median ANI of 99.0, 99.5 and 99.9 %. Although the median ANI values across these datasets were not very close to 100 %, the more clonal clades in the dataset contained very little genetic diversity. As expected, the true tree was reconstructed less accurately when simulated genomes were more similar (Fig. S3). The workflows that showed low Kendall–Colijn metrics between reconstructed phylogenies and the true tree showed a similar pattern in the clonal datasets, although differences are less clear than in Fig. 2.

### *De novo* assembly algorithms have a strong influence on the accuracy of phylogenetic reconstruction

**Fig. 3.**
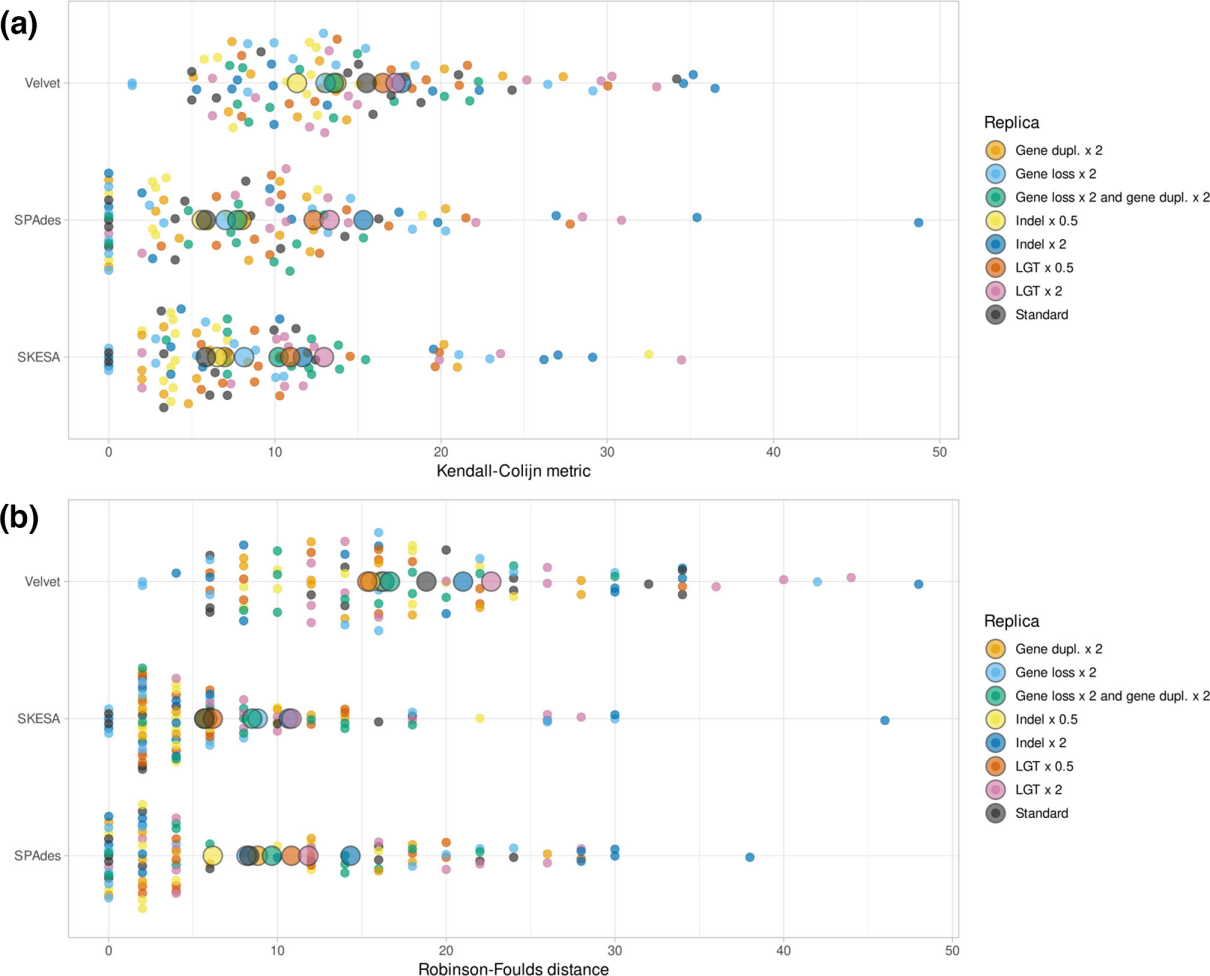
Kendall–Colijn metrics and Robinson–Foulds distances per *de novo* assembly algorithm used in workflows, across eight simulations. Displayed distances are calculated between the ground truth phylogeny and the phylogeny produced by the relevant workflow. Generated using SuperPlotsOfData, and ordered alphabetically. Large circles indicate the median of replicates. Small circles indicate separate measurements for a replica.

Next, we compared the accuracy of phylogenetic reconstruction between workflows employing different *de novo* assembly algorithms (Fig. 3, Table S5). Across eight simulations, workflows employing SPAdes and skesa both resulted in significantly lower Kendall–Colijn metric values compared to the same workflows employing Velvet. In other words, workflows employing SPAdes and skesa reconstruct phylogenies more accurately than the same workflows employing Velvet.

To gain insights in the *de novo* genome assembly quality, we compared the assemblies produced by Velvet, skesa and SPAdes to the *in silico* evolved genomes from which the sequencing reads were generated, using detailed assembly quality metrics such as total genome fraction, NGA50 (N50 of all blocks correctly aligned to the reference genome and corrected for reference genome length) [22], and the number of misassemblies alongside standard quality metrics such as number of contigs or total assembly size. We observed that although Velvet produced genome assemblies with a relatively high NGA50, Velvet also produced the highest number of misassemblies compared to skesa or SPAdes (Fig. S4). SPAdes seemed to perform best across multiple assembly quality metrics, reconstructing a large part of the original genome in few contigs (NGA50, genome fraction reconstructed, number of contigs), with a low number of errors (number of misassemblies).

### Accuracy of phylogenetic reconstruction is associated with the number of informative sites in the alignment

**Fig. 4.**
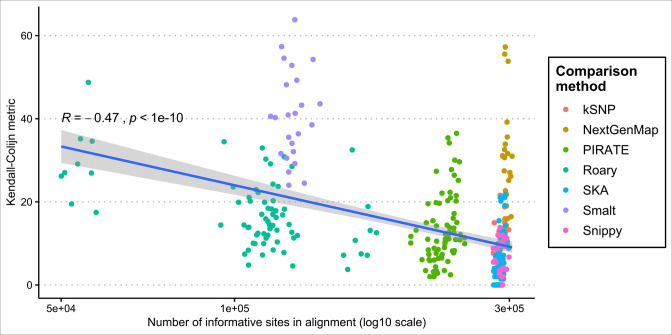
Count of informative sites in the alignment plotted against Kendall–Colijn metric, with a linear model fitted (shading indicates 95 % confidence interval). Pearson’s rho and associated *P* value are shown.

We hypothesized that the workflows using a larger part of the genome in the comparative analysis would yield larger alignments and more accurate phylogenetic reconstruction. To assess this, we extracted the alignment length produced per workflow. We found that the alignment length shows a strong negative correlation with the Kendall–Colijn metric and explains approximately 22 % of variance in the metric (*R* ^2^; Fig. 4). This indicates that the methods that included a larger fraction of the genomes under study produced more accurate phylogenies. When the workflows employing MLST alignments were included, this negative correlation was even stronger (Fig. S5).

### Phylogenetic benchmarking shows a high variability between replicates

**Fig. 5.**
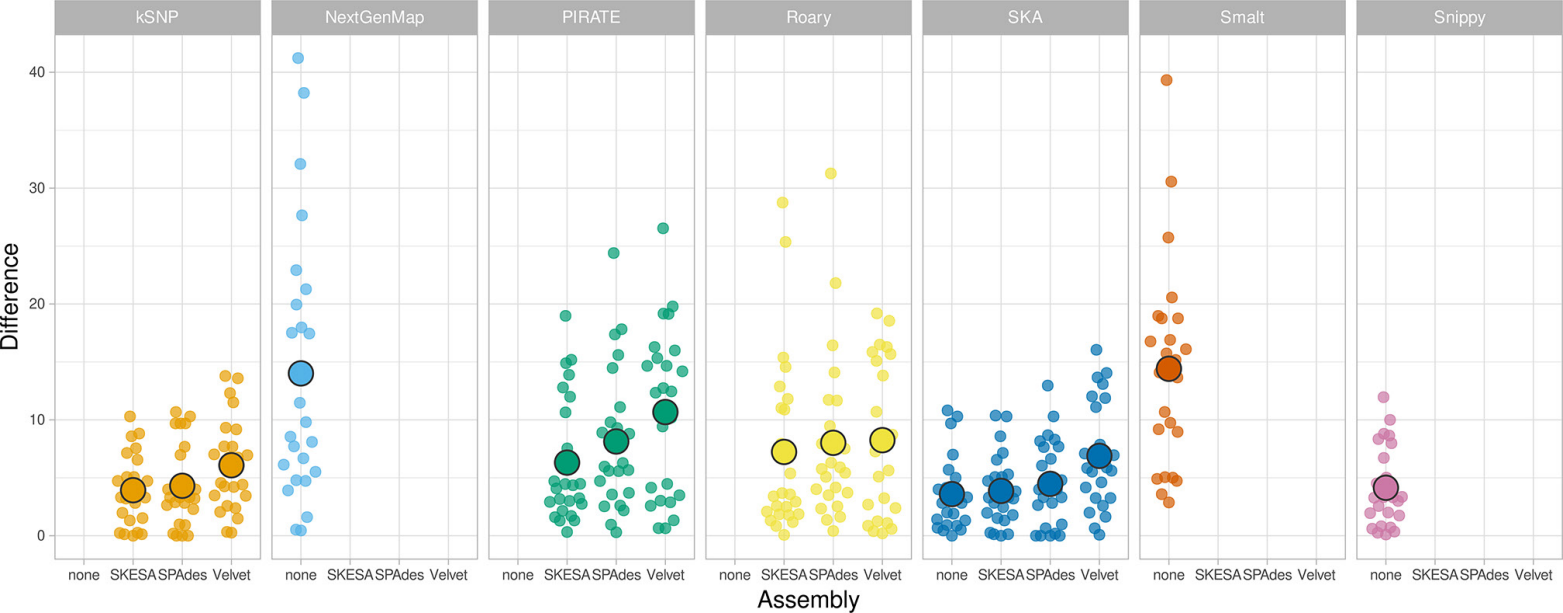
Differences between technical replicates for identical workflows across identical simulations, only differing in starting seed for the simulation. Workflows including MLST were excluded. Generated using SuperPlotsOfData.

Repeating each of the eight simulations three times allows us to assess the reproducibility of this analysis. We see extensive variability in the accuracy of phylogenetic reconstruction even when comparing identical workflows across identical simulations, where only the starting seed for simulation differed (Fig. 5). The largest difference between technical replicates reached a 31 point different in the Kendall–Colijn metric (SPAdes+Roary, simulation with double indel rate). Over 22 % of Kendall–Colijn metric calculations were off more than 10 points between technical replicates.

## Discussion

We have presented a systematic analysis of the accuracy of the phylogenetic reconstruction of several workflows, based on simulated bacterial whole-genome data. We have included 19 phylogenetic workflows. These were each benchmarked using eight simulation scenarios with three independent replicates.

First, we showed that *k*-mer alignment methods provide a good alternative to reference-based mapping in species-level phylogenetic reconstruction. The high accuracy of workflows employing *k*-mer alignment seems to be due to the large fraction of genomes that can be utilized in these workflows, reflected by the high number of informative sites in alignments produced by *k*-mer methods. In more clonal datasets, *k*-mer alignment methods also performed well. Through including eight simulation scenarios, we were able to determine a clear influence of the *de novo* assembly algorithm on phylogenetic accuracy. Based on assembly quality evaluation, we hypothesize that an increased rate of misassemblies has a detrimental effect on phylogenetic accuracy. This also applies to *k*-mer alignment methods, which performed best when combined with either SPAdes or skesa.

Surprisingly, we observed a high variability between replicates of phylogenetic workflows. Over one-fifth of comparisons showed differences of 10 points or more in the Kendall–Colijn metric. To contextualize, the difference in the median Kendall–Colijn metric between the best and worst workflows in Fig. 2(a) was 14.6 points. Generally, workflows using core-gene-alignment methods such as Roary or pirate displayed the highest discrepancies between replicates. This might be because core-gene-alignment methods need to employ heuristics to compare genes in an all-versus-all manner, which could introduce variability in their results.

Across 19 phylogenetic workflows, eight simulations and three replicates, we reconstructed a total of 456 phylogenies. By including multiple workflows, simulations and replicates, this number increases quickly. We were able to limit computational workload by selecting only a single method (iq-tree) to infer phylogenies from alignments. We chose to include only iq-tree because there was little difference between iq-tree, RAxML or other approaches in earlier studies [3]; because iq-tree is widely used and, thus, represents an established method to infer phylogenies; and finally because iq-tree offers the identification of an optimal substitution model through ModelFinder.

We included three reference-mapping tools in this study (Snippy, NextGenMap and smalt). While Snippy performed very well, NextGenMap and smalt performed worse than expected, based on results from a recent benchmark [14]. Several reasons could explain these discrepant findings. First, it should be noted that the previous study primarily aimed to benchmark SNP calling [14], while our study aims to benchmark phylogenetic reconstruction in the presence of various mutational events (including indels, lateral gene transfer, gene rearrangements). Secondly, the genetic distance of our dataset is more comparable with the genetic diversity of the data used in Table 2 and Figure 2 of the recent SNP calling benchmark publication [14]. In that study, NextGenMap and smalt have only been tested on a dataset with a larger genetic distance between reference and sample, hindering direct comparisons with our study. Thirdly, this benchmark has shown NextGenMap and smalt have a lower SNP calling specificity than Snippy [14]. Perhaps for phylogenetic reconstruction, the specificity of SNP calling is more important than the sensitivity. In any case, readers should be cautioned that the results of the specifically for NextGenMap and smalt were unexpected and might not fully reflect the performance of these tools in other settings.

One of the challenges in benchmarking studies is to employ all methods in such a way that these can be compared sensibly. For *k*-mer alignment methods ska and kSNP, we observed that configuring the desired *k*-mer length differs between tools. To obtain aligned *k*-mers of 31 bp, ska requires one to set *k*-mer length (flag ‘-k’) to 15, resulting in the alignment of two split *k*-mers of 15 bp with a middle base, amounting to a total aligned *k*-mer of 31 bp. However, for kSNP the *k*-mer length (flag ‘-k’) should be set to 31, to obtain a 31 bp aligned *k*-mers of which the middle base may vary. Configuring the *k*-mer length correctly resulted in a highly similar accuracy of ska and kSNP, while previous studies did not establish similar performance due to discrepancies in *k*-mer length configuration [25].

Determining the exact rates of genetic events such as point mutations or indels is challenging. In this study, we have evolved bacterial genomes across a range of evolutionary scenarios, which means our results should be interpreted as generalizable findings, rather than findings specific to *E. coli* and its evolutionary mechanisms.

Here, we simulated datasets that exhibited a limited genetic diversity, similar to the genetic diversity observed within species (at least ~95 % ANI) [44]. In the context of more diverse datasets, for example comparing different species or genera, we expect that *k*-mer alignment methods would perform worse, as these methods typically perform best with limited genetic diversity [25]. In accord with our results, we theorize that this is due to a faster decrease in informative sites with increasing evolutionary distance.

The current study focuses on the analysis of short sequencing reads specifically. However, previous studies have investigated the applicability of long-read sequencing (especially Oxford Nanopore Technologies) for outbreak analysis [45, 46]. Analysing long-read sequence data uses fundamentally different algorithms and approaches than short-read sequence data analysis. Future studies could focus on the parameters that influence the accuracy of phylogenetic reconstruction based on long-read sequence data.

This study illustrates how phylogenetic reconstruction methods based on bacterial whole-genome data compare. The simulations cover diverse evolutionary scenarios for bacterial species, providing detailed insight into the performance of phylogenetic reconstruction methods valid across diverse sets of bacterial strains. Recently developed *k*-mer alignment methods achieved similar accuracy as the gold standard (reference mapping) and, thus, seem to be a useful alternative when no suitable reference genome is available. Every microbe evolves according to different evolutionary parameters, so phylogenetic workflows need to be able to resolve many different evolutionary scenarios. Our study provides data on the accuracy of existing phylogenetic workflows and a framework to assess future phylogenetic workflows.

## Supplementary Data

Supplementary material 1

Supplementary material 2

## References

[1] Harris SR, Feil EJ, Holden MTG, Quail MA, Nickerson EK (2010). Evolution of MRSA during hospital transmission and intercontinental spread. Science.

[2] Quainoo S, Coolen JPM, van Hijum SAFT, Huynen MA, Melchers WJG (2017). Whole-genome sequencing of bacterial pathogens: the future of nosocomial outbreak analysis. Clin Microbiol Rev.

[3] Lees JA, Kendall M, Parkhill J, Colijn C, Bentley SD (2018). Evaluation of phylogenetic reconstruction methods using bacterial whole genomes: a simulation based study. Wellcome Open Res.

[4] Timme RE, Rand H, Shumway M, Trees EK, Simmons M (2017). Benchmark datasets for phylogenomic pipeline validation, applications for foodborne pathogen surveillance. PeerJ.

[5] Ahrenfeldt J, Skaarup C, Hasman H, Pedersen AG, Aarestrup FM (2017). Bacterial whole genome-based phylogeny: construction of a new benchmarking dataset and assessment of some existing methods. BMC Genomics.

[6] Hedge J, Wilson DJ (2014). Bacterial phylogenetic reconstruction from whole genomes is robust to recombination but demographic inference is not. mBio.

[7] McTavish EJ, Pettengill J, Davis S, Rand H, Strain E (2017). TreeToReads – a pipeline for simulating raw reads from phylogenies. BMC Bioinformatics.

[8] Nell LA (2020). jackalope: a swift, versatile phylogenomic and high-throughput sequencing simulator. Mol Ecol Resour.

[9] Escalona M, Rocha S, Posada D (2018). NGSphy: phylogenomic simulation of next-generation sequencing data. Bioinformatics.

[10] Saber MM, Shapiro BJ (2020). Benchmarking bacterial genome-wide association study methods using simulated genomes and phenotypes. Microb Genom.

[11] Davín AA, Tricou T, Tannier E, de Vienne DM, Szöllősi GJ (2020). Zombi: a phylogenetic simulator of trees, genomes and sequences that accounts for dead linages. Bioinformatics.

[12] Dalquen DA, Anisimova M, Gonnet GH, Dessimoz C (2012). ALF – a simulation framework for genome evolution. Mol Biol Evol.

[13] Cartwright RA (2005). DNA assembly with gaps (Dawg): simulating sequence evolution. Bioinformatics.

[14] Bush SJ, Foster D, Eyre DW, Clark EL, De Maio N (2020). Genomic diversity affects the accuracy of bacterial single-nucleotide polymorphism-calling pipelines. Gigascience.

[15] Mölder F, Jablonski KP, Letcher B, Hall MB, Tomkins-Tinch CH (2021). Sustainable data analysis with Snakemake. F1000Res.

[16] Kremer PHC, Lees JA, Koopmans MM, Ferwerda B, Arends AWM (2017). Benzalkonium tolerance genes and outcome in *Listeria monocytogenes* meningitis. Clin Microbiol Infect.

[17] Seemann T (2014). Prokka: rapid prokaryotic genome annotation. Bioinformatics.

[18] Huang W, Li L, Myers JR, Marth GT (2012). ART: a next-generation sequencing read simulator. Bioinformatics.

[19] Zerbino DR, Birney E (2008). Velvet: algorithms for de novo short read assembly using de Bruijn graphs. Genome Res.

[20] Bankevich A, Nurk S, Antipov D, Gurevich AA, Dvorkin M (2012). SPAdes: a new genome assembly algorithm and its applications to single-cell sequencing. J Comput Biol.

[21] Souvorov A, Agarwala R, Lipman DJ (2018). SKESA: strategic k-mer extension for scrupulous assemblies. Genome Biol.

[22] Gurevich A, Saveliev V, Vyahhi N, Tesler G (2013). QUAST: quality assessment tool for genome assemblies. Bioinformatics.

[23] Jain C, Rodriguez-R LM, Phillippy AM, Konstantinidis KT, Aluru S (2018). High throughput ANI analysis of 90K prokaryotic genomes reveals clear species boundaries. Nat Commun.

[24] Gardner SN, Slezak T, Hall BG (2015). kSNP3.0: SNP detection and phylogenetic analysis of genomes without genome alignment or reference genome. Bioinformatics.

[25] Harris (2018). SKA: split kmer analysis toolkit for bacterial genomic epidemiology. bioRxiv.

[26] Sedlazeck FJ, Rescheneder P, von Haeseler A (2013). NextGenMap: fast and accurate read mapping in highly polymorphic genomes. Bioinformatics.

[27] Ning Z, Cox AJ, Mullikin JC (2001). SSAHA: a fast search method for large DNA databases. Genome Res.

[28] Page AJ, Cummins CA, Hunt M, Wong VK, Reuter S (2015). Roary: rapid large-scale prokaryote pan genome analysis. Bioinformatics.

[29] Bayliss SC, Thorpe HA, Coyle NM, Sheppard SK, Feil EJ (2019). PIRATE: a fast and scalable pangenomics toolbox for clustering diverged orthologues in bacteria. Gigascience.

[30] Page JA, Taylor B, Keane JA (2016). Multilocus sequence typing by blast from de novo assemblies against PubMLST. J Open Source Softw.

[31] Sievers F, Wilm A, Dineen D, Gibson TJ, Karplus K (2011). Fast, scalable generation of high-quality protein multiple sequence alignments using Clustal Omega. Mol Syst Biol.

[32] Minh BQ, Schmidt HA, Chernomor O, Schrempf D, Woodhams MD (2020). IQ-TREE 2: new models and efficient methods for phylogenetic inference in the genomic era. Mol Biol Evol.

[33] Kalyaanamoorthy S, Minh BQ, Wong TKF, von Haeseler A, Jermiin LS (2017). ModelFinder: fast model selection for accurate phylogenetic estimates. Nat Methods.

[34] Paradis E, Schliep K (2019). ape 5.0: an environment for modern phylogenetics and evolutionary analyses in R. Bioinformatics.

[35] Jombart T, Kendall M, Almagro-Garcia J, Colijn C (2017). treespace: statistical exploration of landscapes of phylogenetic trees. Mol Ecol Resour.

[36] Page AJ, Taylor B, Delaney AJ, Soares J, Seemann T (2016). *SNP-sites*: rapid efficient extraction of SNPs from multi-FASTA alignments. Microb Genom.

[37] Team T pandas development (2019). Pandas-dev/pandas: pandas. Zenodo.

[38] Wickham H, Averick M, Bryan J, Chang W, McGowan L (2019). Welcome to the Tidyverse. J Open Source Softw.

[39] Wickham H (2016). ggplot2: Elegant Graphics for Data Analysis.

[40] Arnold JB (2017). ggthemes: Extra Themes, Scales and Geoms for “ggplot2”, R package version.

[41] Pedersen TL (2017). patchwork: the Composer of ggplots, R package version 00. https://patchwork.data-imaginist.com/reference/patchwork-package.html#author.

[42] Goedhart J (2021). SuperPlotsOfData – a web app for the transparent display and quantitative comparison of continuous data from different conditions. Mol Biol Cell.

[43] Jones E, Oliphant T, Peterson P (2001). SciPy: Open Source Scientific Tools for Python. http://www.scipy.org/.

[44] Chun J, Oren A, Ventosa A, Christensen H, Arahal DR (2018). Proposed minimal standards for the use of genome data for the taxonomy of prokaryotes. Int J Syst Evol Microbiol.

[45] Greig DR, Jenkins C, Gharbia SE, Dallman TJ (2021). Analysis of a small outbreak of Shiga toxin-producing *Escherichia coli* O157:H7 using long-read sequencing. Microb Genom.

[46] Quick J, Ashton P, Calus S, Chatt C, Gossain S (2015). Rapid draft sequencing and real-time nanopore sequencing in a hospital outbreak of *Salmonella*. Genome Biol.

[47] Wick RR, Schultz MB, Zobel J, Holt KE (2015). Bandage: interactive visualization of de novo genome assemblies. Bioinformatics.

