# Supplementary material for: Benchmarking the topological accuracy of bacterial phylogenomic workflows using in silico evolution
Source: Microb Genom. 2022 Mar 15;8(3):000799. doi: 10.1099/mgen.0.000799 (PMC9176278; doi:10.1099/mgen.0.000799)
Supplement: Supplementary material 1 [file mgen-8-0799-s001.pdf]

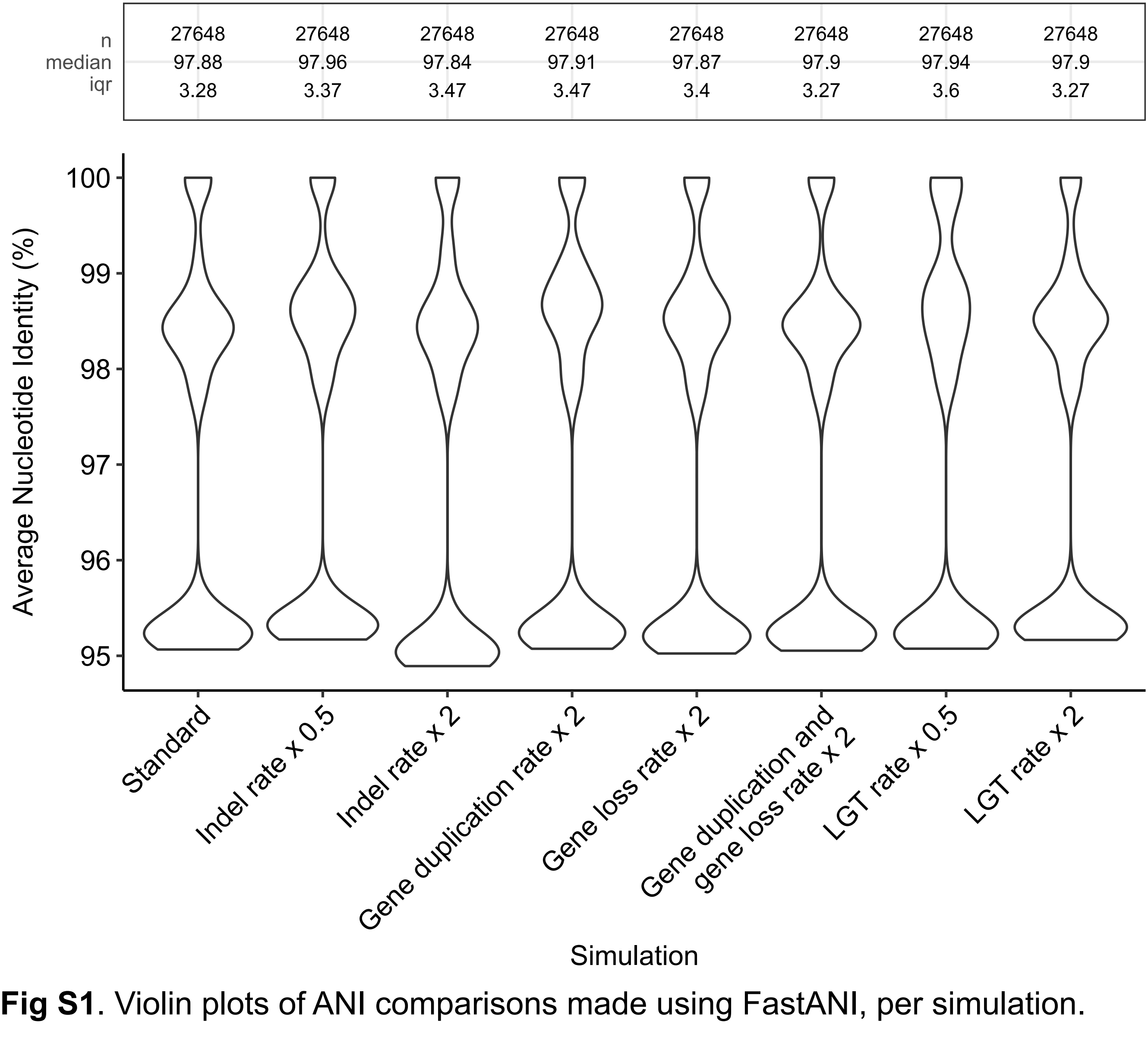

Fig S1. Violin plots of ANI comparisons made using FastANI, per simulation. From each simulation replicate (three replicates for eight simulations), all 96 *in silico* evolved genomes were compared in an all vs. all fashion.

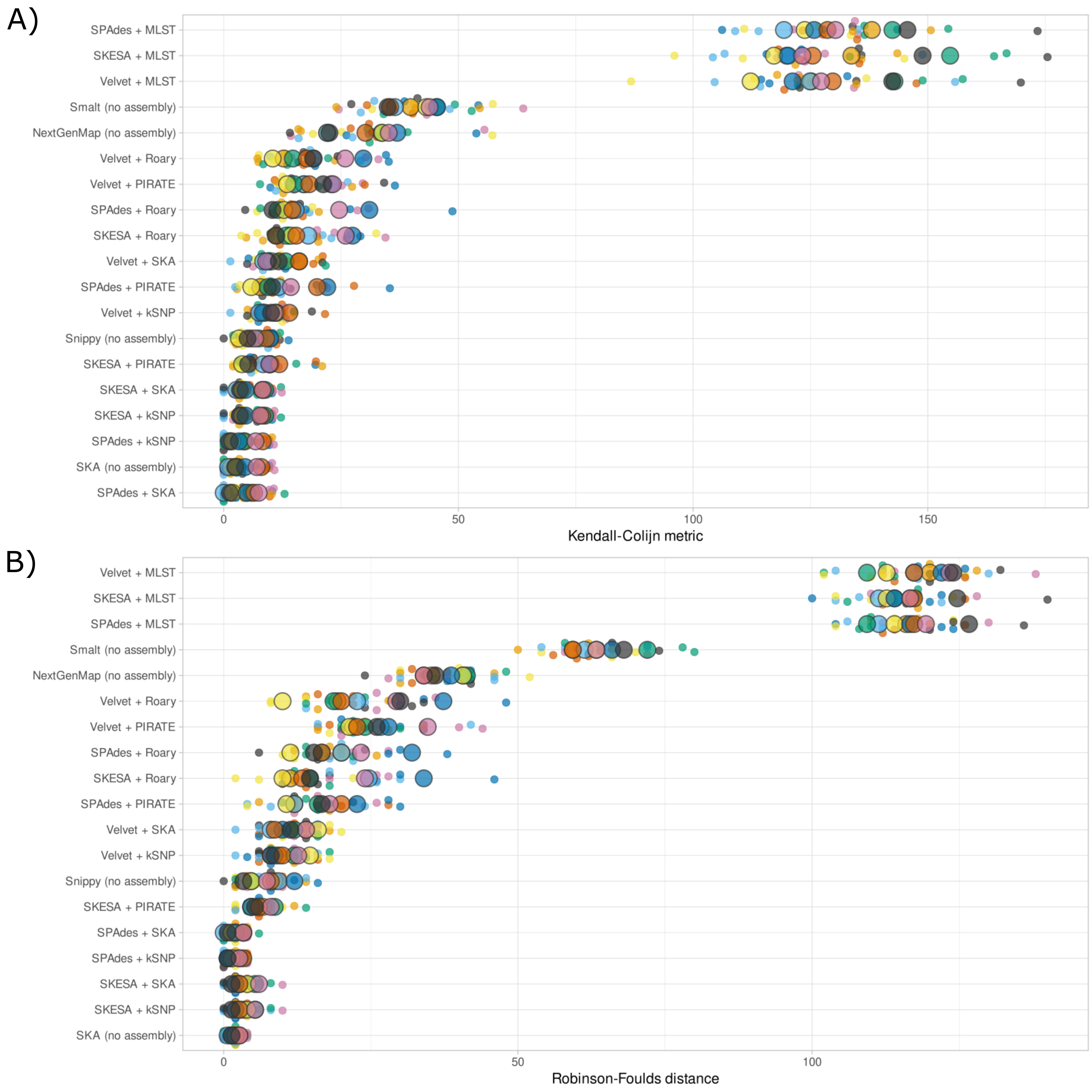

Figure S2. Kendall-Colijn metrics and Robinson-Foulds distances between the ground truth phylogeny and phylogenies produced by workflows, across eight simulations.

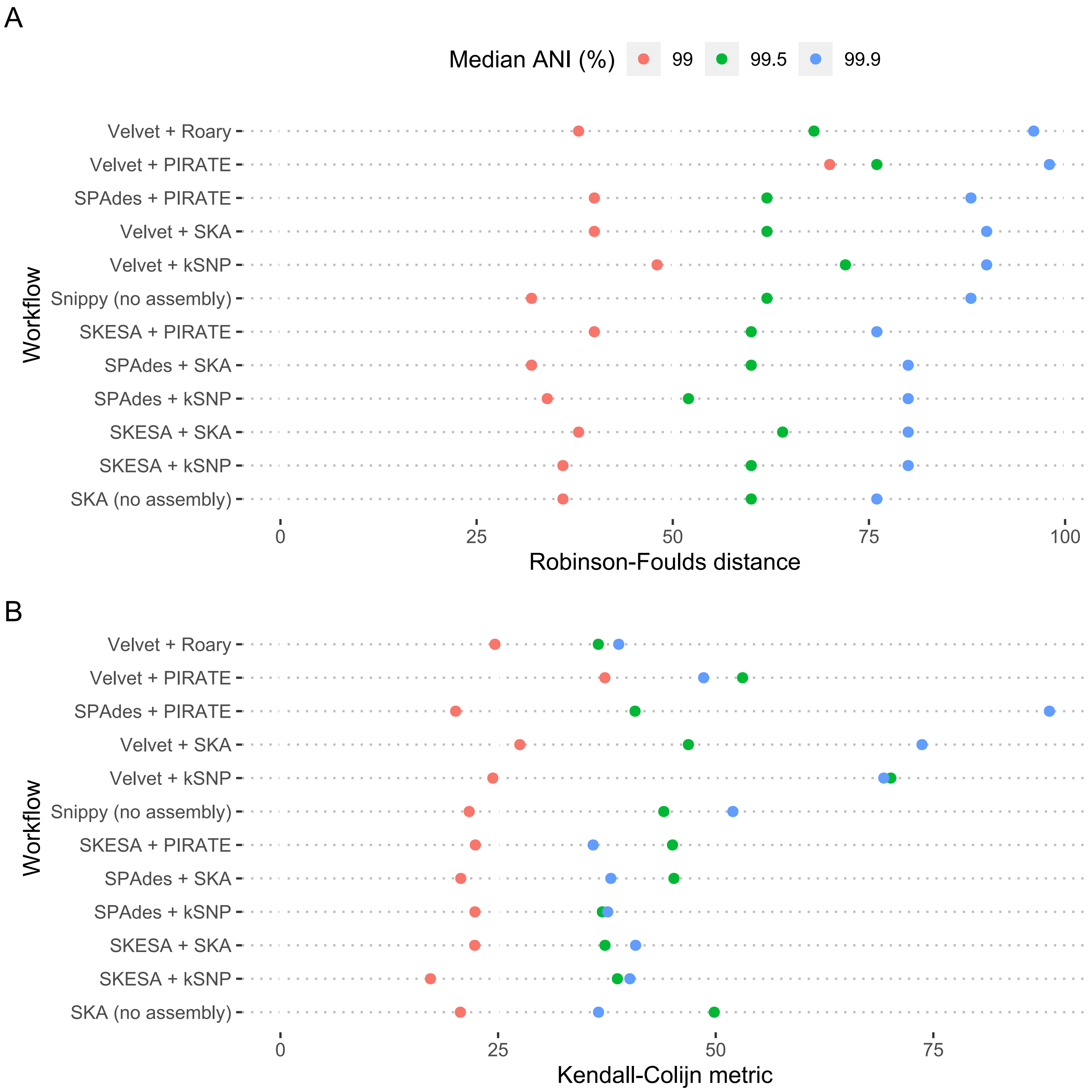

Fig S3. Kendall-Colijn metrics and Robinson-Foulds distances per phylogenetic workflow for clonal simulated data. Displayed distances are calculated between the ground truth phylogeny and the phylogeny produced by the relevant workflow.

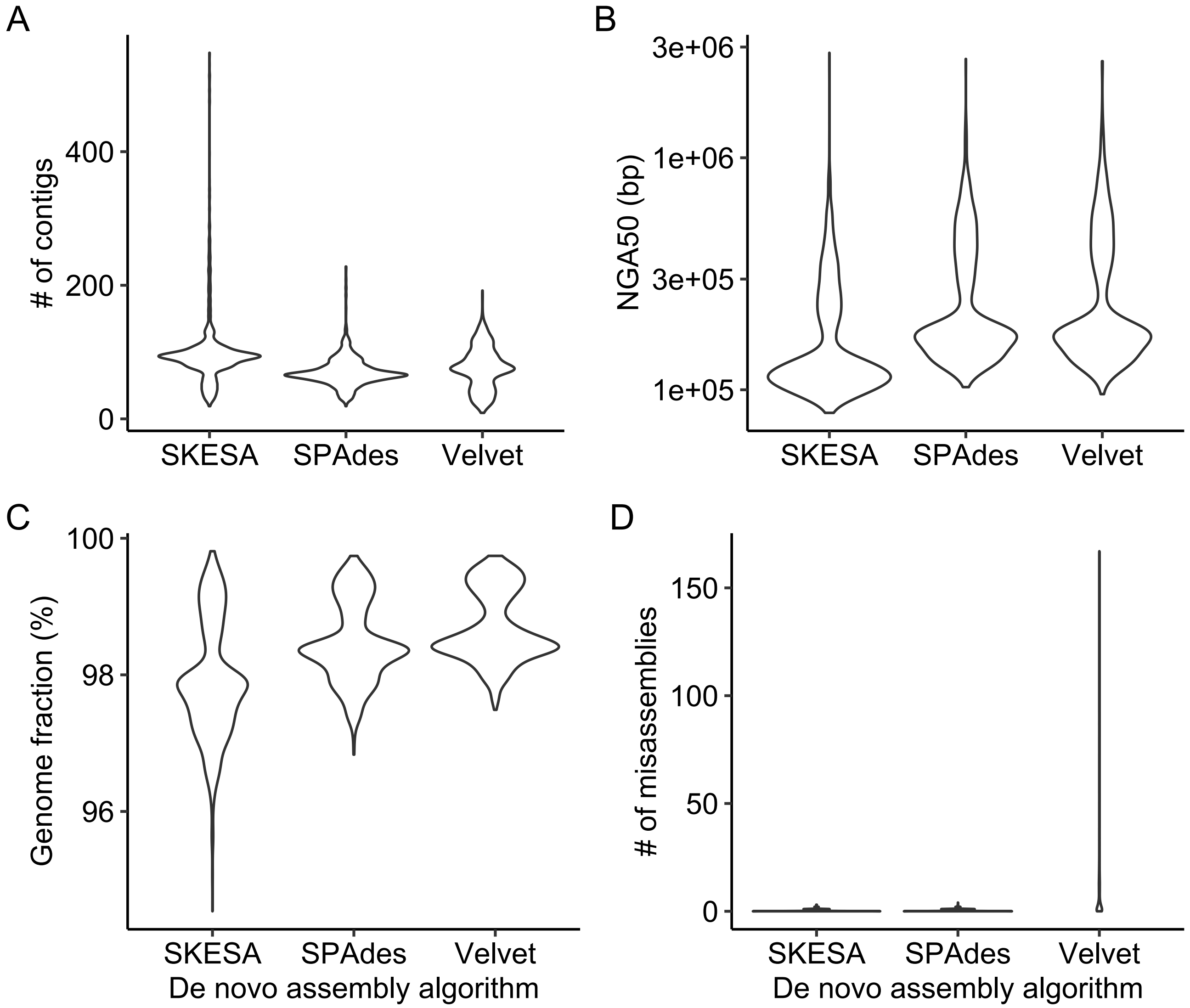

Figure S4. Comparison of SKESA, SPAdes and Velvet algorithms for *de novo* genome assembly, based on number of contigs, NGA50, genome fraction reconstructed and number of misassemblies.

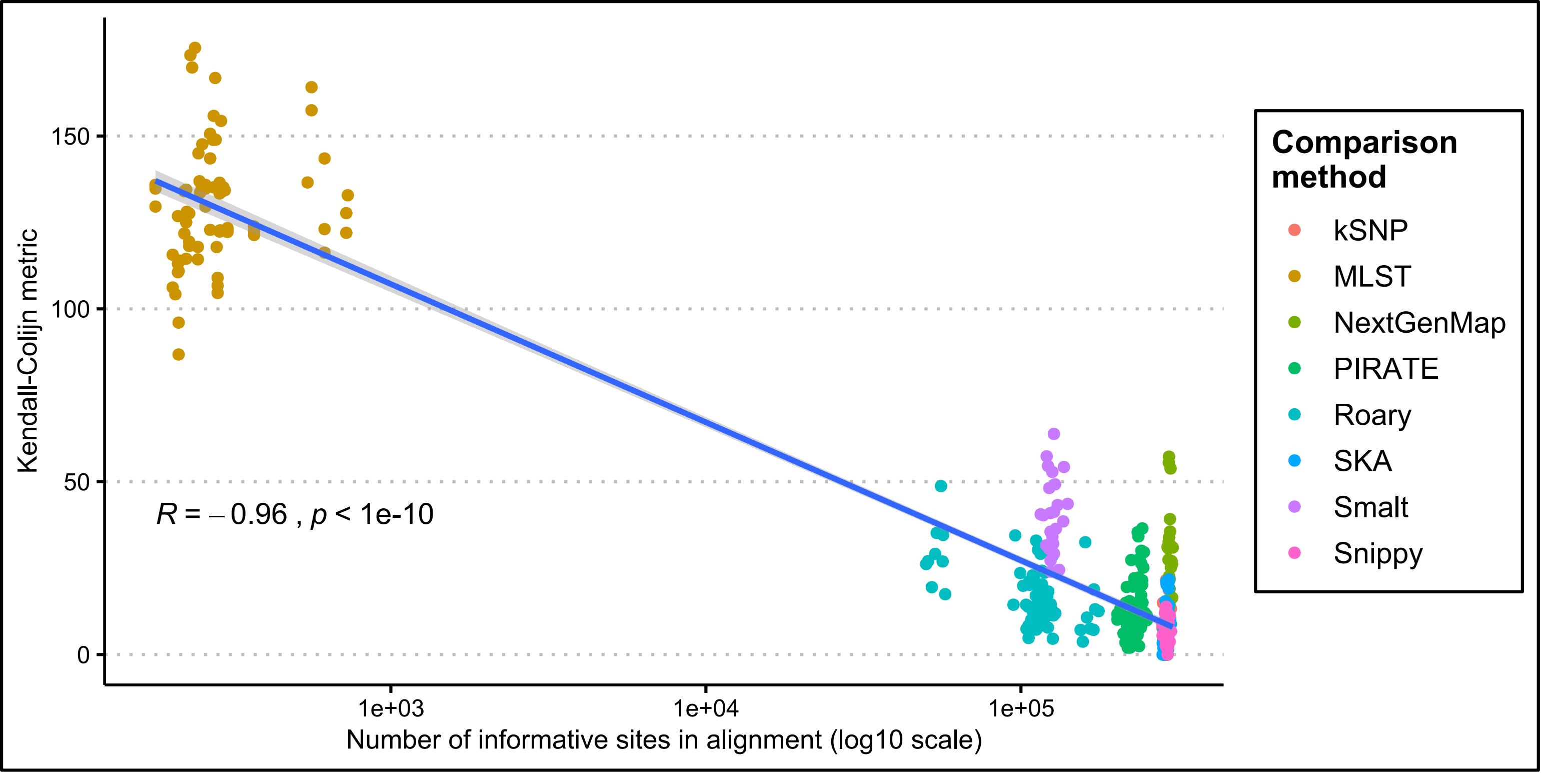

Figure S5. Count of informative sites in alignment plotter against Kendall-Colijn metric, with a linear model fitted (shading indicates 95% confidence interval). Pearson’s Rho and associated p-value are shown.
